# Subgroups based on autoantibody status associated with clinical manifestations, *HLA-DRB1* variants, cytokines, and flare of vasculitis in childhood-onset systemic lupus erythematosus

**DOI:** 10.3389/fimmu.2026.1766478

**Published:** 2026-03-16

**Authors:** Shengfang Bao, Jingyi Lu, Jiani Ma, Yingying Jin, Hua Huang, Zhen Yang, Xuemei Xu, Chenxi Liu, Xiqiong Han, Liping Wang, Sun Chen, Yufeng Li, Yanliang Jin

**Affiliations:** 1 Department of Rheumatology and Immunology, Shanghai Children’s Medical Center, School of Medicine, Shanghai Jiao Tong University, Shanghai, China; 2 Department of Pediatric Cardiology, Xinhua Hospital, School of Medicine, Shanghai Jiao Tong University, Shanghai, China; 3 Department of Pediatric Nephrology, Rheumatology and Immunology, Xinhua Hospital, School of Medicine, Shanghai Jiao Tong University, Shanghai, China

**Keywords:** belimumab, cluster analysis, cytokines, HLA, interferon-alpha, interleukin, lupus

## Abstract

**Objective:**

Childhood-onset systemic lupus erythematosus (cSLE) exhibits significant heterogeneity, leading to challenges in prognosis and treatment. This study aims to stratify cSLE patients into clinically distinct subgroups based on routine autoantibody profiles and to characterize these subgroups by their differences in *HLA-DRB1* genotypes, cytokine signatures, clinical manifestations, and flare incidence.

**Methods:**

We conducted a retrospective study of 102 cSLE patients. An unsupervised two-step cluster analysis was performed using nine routinely measured autoantibodies. The resulting subgroups were compared for clinical features, *HLA-DRB1* allele frequencies, serum cytokine levels, and flare-free survival using Kaplan–Meier analysis.

**Results:**

Cluster analysis identified two distinct subgroups. Subgroup 2, characterized by anti-Sm/RNP positivity, demonstrated significantly more severe disease, including higher rates of lupus nephritis, neuropsychiatric involvement, and elevated SLE disease activity index (SLEDAI) scores at diagnosis compared to subgroup 1 (anti-Sm/RNP-negative). Genetically, subgroup 2 was enriched with the HLA-DRB1*15 and *09 alleles. Immunologically, subgroup 2 exhibited significantly elevated IFN-α levels. Despite more frequent use of belimumab, subgroup 2 had a significantly lower flare-free survival rate than subgroup 1 (*P* < 0.001).

**Conclusion:**

Autoantibody-based stratification effectively delineates two cSLE subgroups with distinct genetic, immunological, and clinical trajectories. The anti-Sm/RNP positive subgroup, defined by HLA-DRB1*15/09 risk alleles and a high IFN-α signature, represents a more severe phenotype with a higher risk of flare, potentially explaining the suboptimal response to belimumab in this group.

## Introduction

Systemic lupus erythematosus (SLE) is a systemic autoimmune disease causing substantial organ damage and failure (1). Approximately 20% of SLE cases manifest before the age of 18 years, leading to a diagnosis of childhood-onset SLE (cSLE). However, cSLE is characterized by multiorgan impairment with considerable heterogeneity, and limited data is available. Although therapeutic advances have markedly improved survival rates in cSLE, severe organ involvement or disease flares continue to contribute to poor long-term outcomes. Thus, further investigation into cSLE remains essential.

Autoantibody testing is routine and necessary in SLE, making it a straightforward basis for patient stratification (2). In our prior research, we demonstrated that stratification by autoantibody profiles helps distinguish phenotypic variations and disease activity (3). Moreover, it has been found that distinct autoantibody patterns reflect the genetically driven mechanisms associated with *HLA-DRB1* alleles in autoimmune diseases (2, 4–6). These alleles encode antigen-presenting molecules that facilitate peptide presentation to T cells, initiating antigen-specific adaptive immune responses that could promote autoantibody production and cytokine release. However, a multidimensional assessment of *HLA-DRB1* alleles, cytokine profiles, and disease flares is absent in autoantibody-based subgroups of cSLE. Therefore, this study aims to investigate differences in *HLA-DRB1* genotypes, cytokines, clinical manifestations, and flare incidence across autoantibody-based subgroups of cSLE in order to elucidate underlying disease mechanisms and to facilitate personalized treatment strategies for specific cSLE populations.

## Methods

### Study population

**Figure 1:**
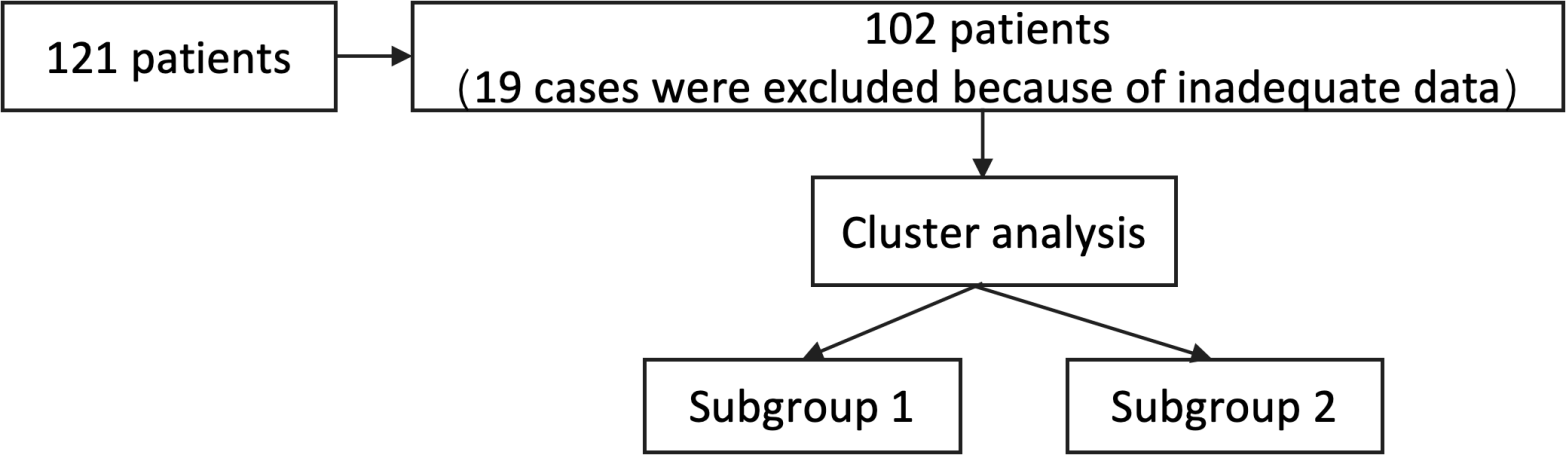
Flowchart of patient cohort selection and subgroup identification via cluster analysis. The initial cohort consisted of 121 patients. Following the exclusion of 19 cases due to inadequate data, 102 patients were included in the subsequent analysis. Cluster analysis was performed on these 102 patients, resulting in the identification of two distinct subgroups.

We conducted a retrospective study of 121 children with SLE who were diagnosed in the Department of Rheumatology & Immunology at Shanghai Children’s Medical Center and Xinhua Hospital from August 2022 to September 2025. Nineteen patients with incomplete medical charts were excluded. There were 102 patients (27 males and 75 females) for final analysis (**Figure 1**). The mean age at diagnosis was 10.93 ± 2.40 years (range: 3.00–16.00 years). The median follow-up time for the cohort was 2.92 years. All patients fulfilled the American College of Rheumatology (ACR) criteria, with disease onset at ages before 18 years.

### Clinical and laboratory data collection

Clinical and laboratory variables were collected retrospectively from medical records at the time of diagnosis. Clinical manifestations were assessed according to the ACR criteria and included mucocutaneous (malar rash, discoid rash, photosensitivity, and oral ulcers), musculoskeletal (arthritis), serosal (pleuritis and pericarditis), lupus nephritis (LN), neuropsychiatric, and hematological involvements.

LN was defined according to the International Society of Nephrology/Renal Pathology Society (ISN/RPS) classification. Only patients with a kidney biopsy confirming LN were included in the analysis. Patients without biopsy or with insufficient pathological data were excluded.

Disease activity was measured using the SLE disease activity index (SLEDAI). Laboratory parameters included complement C3 and C4 levels and anti-dsDNA antibody titers. All these variables were compared between the subgroups.

### Autoantibody data collection

Antibodies were detected by double immunodiffusion (DID) at baseline and prior to immunosuppressive treatment. All tests were performed when blood was drawn according to standard protocol in the department. aPL antibodies, including anti-CL IgG/IgM and anti-β 2GP1 IgG, were analyzed in the serum by ELISA, with aPL cutoff levels for positivity corresponding to the 99th percentile of the normal population (2).

### Genotyping

The *HLA-DRB1* genotyping was performed by the PCR-SBT method and referenced to the IMGT/HLA database. The *HLA-DRB1* allele frequencies for patients were obtained by direct counting. An individual was considered homozygous if only one allele was detected in the genotyping assay, and the allele frequency was counted twice (7).

### Cytokine analysis

All cytokine measurements were performed on baseline serum samples obtained at diagnosis and prior to immunosuppressive treatment. The serum concentrations of IL-1β, IL-2R, IL-4, IL-5, IL-6, IL-8, IL-10, IL-12p70, IL-17A, TNF-α, IFN-α, and IFN-γ were analyzed. A commercially available fluorescence flow cytometry assay kit (MAGPIX, USA) was used, and cytokine concentrations were determined in accordance with the manufacturer’s instructions (8). Samples were run undiluted in triplicate, and the mean value of the three technical replicates was calculated for each sample. Laboratory staff and clinicians were blind to each other.

### Two-step cluster analysis

Two-step cluster, an approach for exploring empirical groups of individuals with similar characteristics, is hypothesis free and uses the log-likelihood distance measure. The optimal number of clusters was automatically determined by the Schwarz Bayesian Information Criterion and the large ratio of distance measures. The final model’s validity was confirmed by the silhouette coefficient (a value > 0.50 was interpreted as good fitting, between 0.30 and 0.50 as fair, and <0.30 as poor) and was also testified to with clinical interpretability. Herein, the two-step cluster analysis was performed on the set of nine autoantibodies [double-stranded DNA (dsDNA), nucleosome, histone, ribosomal P protein, Smith (Sm), u1- ribonucleoprotein (RNP), Sjögren’s syndrome antigen A (SSA)/Ro52, SSA/Ro60, and Sjögren’s syndrome antigen B (SSB)/La], which successfully reproduced the two distinct patient subgroups (silhouette coefficient = 0.4) (**Supplementary Figure 1**), in line with our prior study (3). The addition of antiphospholipid antibodies (aCL and anti-β2GP1) reduced cluster quality (silhouette coefficient = 0.3) and were therefore excluded from the final model.

### Statistical analysis

Continuous variables were expressed as mean ± SD or median and interquartile range (Q1, Q3). Categorical variables were presented as an absolute number (frequency). Two-step cluster analysis was used to identify groups of patients with similar autoantibody profiles. The result of two clusters was interpretable and clinically meaningful. The clusters were referred to as disease subgroups in this study. Logistic regression was performed to assess the association between clinical variables and each subgroup.

To evaluate number (frequency), categorical variable comparisons were first assessed by Pearson’s χ^2^ test, which requires that at least 80% of the cells must have an expected frequency of ≥5 and no cell must have an expected frequency <1. Fisher’s exact test (*n* ≤ 5) was used. Flare-free survival rates of the two subgroups were studied by the Kaplan–Meier method and compared using a log-rank test. All statistical analyses were performed using SPSS V.26.0. The statistical tests were two-sided and a *p* < 0.05 was considered statistically significant.

To minimize bias, the investigators performing the cluster and statistical analyses were blinded to the patients’ treatment assignments and clinical outcomes.

## Result

### Characteristics of the participants in subgroups defined by autoantibody status

Two-step cluster analysis based on autoantibody status grouped patients with cSLE into two subgroups (silhouette coefficient = 0.4). Subgroup 1 was characterized by negative anti-Sm/RNP, and subgroup 2 was dominated by positive anti-Sm/RNP (**Table 1**). The predictor importance derived from the cluster analysis confirmed anti-Sm and anti-RNP as the top contributors, followed by anti-nucleosome, anti-histone, anti-ribosomal P protein, and anti-dsDNA (**Supplementary Figure 1**). Meanwhile, subgroup 2 exhibited a higher frequency of multiple autoantibody positivity than subgroup 1. Subgroup 2 was enriched for ≥5 autoantibodies (58.6% *vs*. 12.5%). The detailed distribution was presented in **Table 1**.

**Table 1:** Characteristics of autoantibody pattern in cSLE patients at diagnosis.

Autoantibody	Subgroup 1 *n* = 32 (31.4%)	Subgroup 2 *n* = 70 (68.6%)
Anti-Sm	0 (0.0%)	61 (87.1%)
Anti-u1-RNP	0 (0.0%)	61 (87.1%)
Anti-dsDNA	7 (21.9%)	36 (51.4%)
Anti-nucleosome	1 (3.1%)	39 (55.7%)
Anti-ribosomal P protein	3 (9.4%)	36 (51.4%)
Anti-histone	2 (6.3%)	38 (54.3%)
Anti-Ro52/SSA	18 (56.3%)	28 (40.0%)
Anti-Ro60/SSA	18 (56.3%)	31 (44.3%)
Anti-La/SSB	13 (40.6%)	14 (20.0%)
Anti-CL IgG	4 (12.5%)	20 (28.6%)
Anti-CL IgM	4 (12.5%)	12 (17.1%)
Anti-β 2GP1 IgG	6 (18.8%)	29 (41.4%)
Anti-β 2GP1 IgM	3 (9.4%)	15 (21.4%)
Number of positive autoantibodies		
3 positive autoantibodies	15 (46.9%)	2 (2.9%)
4 positive autoantibodies	13 (40.6%)	27 (38.6%)
≥5 positive autoantibodies	4 (12.5%)	41 (58.6%)

There were no significant differences in age and gender between subgroups. LN was significantly more frequent in subgroup 2 than in subgroup 1 [*p* = 0.003, OR 5.56 (95% CI 1.76–17.55)]. The detailed distribution of ISN/RPS classes was shown in **Table 2**.

**Table 2:** Characteristics of subgroups of cSLE patients.

Variable	Subgroup 1 *n* = 32 (31.4%)	Subgroup 2 *n* = 70 (68.6%)	*P*-value	OR (95% CI)
Age	10.931 ± 2.738	10.910 ± 2.274	0.686	
Gender (female, %)	21 (65.6%)	54 (77.1%)	0.221	
SLEDAI	7.094 ± 6.229	12.257 ± 6.648	0.001	1.15 (1.06–1.25)
Lupus nephritis, *n* (%)	4 (12.5%)	31 (44.3%)	0.003	5.56 (1.76–17.55)
ISN/RPS class, *n* (%)*				
-Class II	2(50.0%)	0		
-Class III	2(50.0%)	7 (22.6%)		
-Class IV	0	8 (25.8%)		
-Class V	0	4 (12.9%)		
-Class III + V	0	5 (16.1%)		
-Class IV + V	0	7 (22.6%)		
Neuropsychiatric lupus, *n* (%)	2 (6.3%)	17 (24.3%)	0.044	4.81 (1.04–22.27)
Serositis, *n* (%)	5 (15.6%)	34 (48.6%)	0.003	5.10 (1.76–14.77)
C3(g/L)	0.32 ± 0.11	0.30 ± 0.14	0.214	0.99 (0.96–1.02)
C4(g/L)	0.06 ± 0.02	0.06 ± 0.03	0.638	1.00 (0.85–1.18)
anti-dsDNA(IU/ml)	359.4 ± 138.2	550.7 ± 225.8	0.008	1.01 (1.00–1.01)
Leukopenia, *n* (%)	27 (84.4%)	59 (84.3%)	0.991	0.99 (0.31–3.19)
Autoimmune anemia, *n* (%)	16 (50.0%)	27 (38.6%)	0.280	0.63 (0.27–1.46)
Thrombocytopenia, *n* (%)	8 (25.0%)	16 (22.9%)	0.812	0.89 (0.34–2.35)
Sicca symptoms, *n* (%)	9 (28.1%)	14 (20.0%)	0.365	0.64 (0.24–1.68)
Mucocutaneous manifestations, *n* (%)	22 (68.8%)	58 (82.9%)	0.108	2.20 (0.83–5.85)
Musculoskeletal manifestations, *n* (%)	8 (25.0%)	22 (31.4%)	0.509	1.37 (0.54–3.51)
Belimumab, *n* (%)	13 (40.6%)	49 (70.0%)	0.006	3.41 (1.43–8.15)
Rituximab, *n* (%)	3 (9.4%)	14 (20.0%)	0.180	2.42 (0.64–9.14)

*Data are presented as *n* (%), with percentages calculated based on the number of lupus nephritis patients in each subgroup.

Neuropsychiatric involvement [*p* = 0.044, OR 4.81 (95% CI 1.04–22.27)] and serositis [*p* = 0.003, OR 5.10 (95% CI 1.76–14.77)] were also significantly more common in subgroup 2. In contrast, the frequencies of mucocutaneous manifestations and musculoskeletal manifestations did not differ significantly between the two subgroups (**Table 2**).

Regarding laboratory parameters, anti-dsDNA titers were significantly higher in subgroup 2 (550.7 ± 225.8 IU/ml *vs*. 359.4 ± 138.2 IU/ml, *p* = 0.008). No significant differences were found in C3 or C4 levels, nor in the frequencies of leukopenia, autoimmune anemia, or thrombocytopenia (**Table 2**).

The use of biologic agents was detailed in **Table 2**. Belimumab was initiated according to physician assessment of disease activity and treatment guidelines, with no patient switching to belimumab due to intolerance to prior therapies. Belimumab was required significantly more frequently in subgroup 2 [p = 0.006, OR 3.41 (95% CI 1.43–8.15)]. Rituximab use was also more common in subgroup 2, although the difference did not reach statistical significance (*p* = 0.180).

### *HLA-DRB1* variants among participants within different subgroups of cSLE

We compared the *HLA-DRB1* allele frequencies between the two subgroups of patients with SLE (**Table 3**). The HLA-DRB1*15 allele and the HLA- DRB1*9 allele were more frequent in subgroup 2 compared with subgroup 1. The HLA-DRB1*8 allele, the HLA-DRB1*11 allele, and the HLA-DRB1*12 allele were more frequent in subgroup 1 compared with subgroup 2.

**Table 3:** OR and 95% CI of the *HLA-DRB1* alleles in different subgroups of cSLE patients.

HLA allele	Subgroup 1 *n* = 32 (2*n* = 64)	Subgroup 2 *n* = 70 (2*n* = 140)	*P*-value	OR	95% CI
	AC (AF)	AC (AF)			
Two-digit HLA allele					
DRB1*15	7 (10.9%)	39(27.9%)	0.010	3.144	1.320–7.487
DRB1*09	6 (9.4%)	30(21.4%)	0.042	2.636	1.038–6.698
DRB1*08	13(20.3%)	4 (2.9%)	<0.001	0.115	0.036–0.370
DRB1*07	8 (12.5%)	22(15.7%)	0.548	1.305	0.547–3.113
DRB1*04	7 (10.9%)	8 (5.7%)	0.192	0.494	0.171–1.426
DRB1*12	7 (10.9%)	5 (3.6%)	0.048	0.302	0.092–0.990
DRB1*11	5 (7.8%)	1 (0.7%)	0.026	0.085	0.010–0.742
DRB1*16	4 (6.3%)	11 (7.9%)	0.684	1.279	0.391–4.182
DRB1*13	3 (4.7%)	7 (5.0%)	0.924	1.070	0.268–4.280
DRB1*14	2 (3.1%)	4 (2.9%)	0.916	0.912	0.163–5.111
DRB1*3	1 (1.6%)	8 (5.7%)	0.211	3.818	0.467–31.193
DRB1*1	1 (1.6%)	1 (0.7%)	0.578	0.453	0.028–7.363
Four-digit HLA allele	Subgroup 1 *n* = 32 (2*n* = 64)	Subgroup 2 *n* = 70 (2*n* = 140)			
DRB1*15:01	6 (9.4%)	32 (22.9%)	0.026	2.864	1.132–7.248
DRB1*15:02	1 (1.6%)	7 (5.0%)	0.267	3.316	0.399–27.531
DRB1*09:01	6 (9.4%)	30(21.4%)	0.042	2.636	1.038–6.698
DRB1*07:01	8 (12.5%)	22(15.7%)	0.548	1.305	0.547–3.113
DRB1*16:02	4 (6.3%)	11 (7.9%)	0.684	1.279	0.391–4.182
DRB1*04:06	4 (6.3%)	2 (1.4%)	0.083	0.217	0.039–1.219
DRB1*04:05	2 (3.1%)	5 (3.6%)	0.871	1.148	0.217–6.082
DRB1*04:08	1 (1.6%)	1 (0.7%)	0.578	0.453	0.028–7.363
DRB1*03:01	1 (1.6%)	8 (5.7%)	0.211	3.818	0.467–31.193
DRB1*13:02	2 (3.1%)	6 (4.3%)	0.693	1.388	0.272–7.073
DRB1*13:01	1 (1.6%)	1 (1.6%)	0.578	0.453	0.028–7.363
DRB1*12:02	7 (10.9%)	5 (3.6%)	0.048	0.302	0.092–0.990
DRB1*14:54	1 (1.6%)	2 (1.4%)	0.941	0.913	0.081–10.257
DRB1*14:05	1 (1.6%)	2 (1.4%)	0.941	0.913	0.081–10.257
DRB1*8:03	13(20.3%)	4 (2.9%)	<0.001	0.115	0.036–0.370
DRB1*11:01	5 (7.8%)	1 (0.7%)	0.026	0.085	0.010–0.742
DRB1*01:01	1 (1.6%)	1 (0.7%)	0.578	0.453	0.028–7.363

AC, allele count; AF, allele frequency; HLA, human leucocyte antigen; 2*n*, allelic count.

Analysis by four-digit *HLA-DRB1* allele subtypes further revealed that the HLA-DRB1*15:01, DRB1*15:02, and DRB1*09:01 were more frequent in subgroup 2 compared with subgroup 1. The HLA-DRB1*8:03, DRB1*11:01, and DRB1*12:02 were more frequent in subgroup 1 compared with subgroup 2.

Furthermore, the allele frequency of HLA-DRB1*15 was significantly higher in our SLE cohort (22.5%, 46/204) compared to the general population (14.6%, 92/628) as reported in the Allele Frequency Net Database (AFND) (*p* < 0.01).

### Cytokine evaluations among participants with different subgroups of cSLE

To further address SLE heterogeneity, we compared the cytokine levels at diagnosis (prior to the treatment of prednisone or immunosuppressants) between two subgroups of patients with cSLE (**Table 4**). Subgroup 2 exhibited significantly higher IFN-α levels (*p* = 0.020), with a 3.02-fold increase (95% CI 1.20–7.60). In contrast, IL-4 and IL-17A were significantly lower in subgroup 2, with fold changes of 0.32 (95% CI 0.13–0.81) and 0.58 (95% CI 0.36–0.92), respectively.

**Table 4:** Cytokines in subgroups of cSLE patients at diagnosis.

Cytokines (pg/ml)	Subgroup 1 median (Q1–Q3)	Subgroup 2 median (Q1–Q3)	*P*	Fold change (95% CI)
IL-1b	2.5 (2.5–3.4)	4.2 (0.0–7.0)	0.073	1.48 (0.96–2.28)
IL-2R	154.7 (10.1–276.8)	152.3 (12.0–437.0)	0.132	1.54 (0.87–2.72)
IL-4	14.3 (4.4–61.4)	5.0 (0.0–5.8)	0.018	0.32 (0.13–0.81)
IL-5	3.6 (3.6–4.5)	3.6 (2.5–6.1)	0.202	1.21 (0.90–1.62)
IL-6	2.9 (2.5–10.9)	3.5 (2.2–19.3)	0.084	1.85 (0.92–3.72)
IL-8	13.2 (8.7–48.6)	29.8 (6.0–72.1)	0.158	1.52 (0.85–2.73)
IL-10	4.4 (0.0–7.8)	5.9 (0.0–13.8)	0.404	1.31 (0.69–2.49)
IL-12P70	3.1 (2.5–4.2)	2.5 (2.5–4.4)	0.124	0.91 (0.67–1.24)
IL-17A	10.8 (8.1–21.2)	6.0 (0.0–11.8)	0.020	0.58 (0.36–0.92)
IL-18	8.7 (0.0–50.0)	8.0 (0.0–16.0)	0.926	0.98 (0.58–1.65)
IFN-α	3.9 (0.0–7.4)	6.5 (4.3–104.5)	0.020	3.02 (1.20–7.60)
IFN-γ	6.6 (3.9–7.9)	7.3 (0.0–9.4)	0.059	1.83 (0.98–3.42)
TNF-α	4.1 (3.3–4.1)	5.3 (4.0–8.5)	0.137	1.27 (0.93–1.72)

### Flare in subgroups

We also evaluated the incidence of flares in subgroups of cSLE patients. The etiologies of flare in subgroup 2 were analyzed and attributed to the following: progression to LN in patients without initial renal presentation (*n* = 10); infection-precipitated flares (*n* = 9), manifesting as transient proteinuria; non-adherence to belimumab (*n* = 6), with subsequent disease control upon drug reintroduction; and relapses despite standard treatment without detectable infection (*n* = 6).

**Figure 2:**
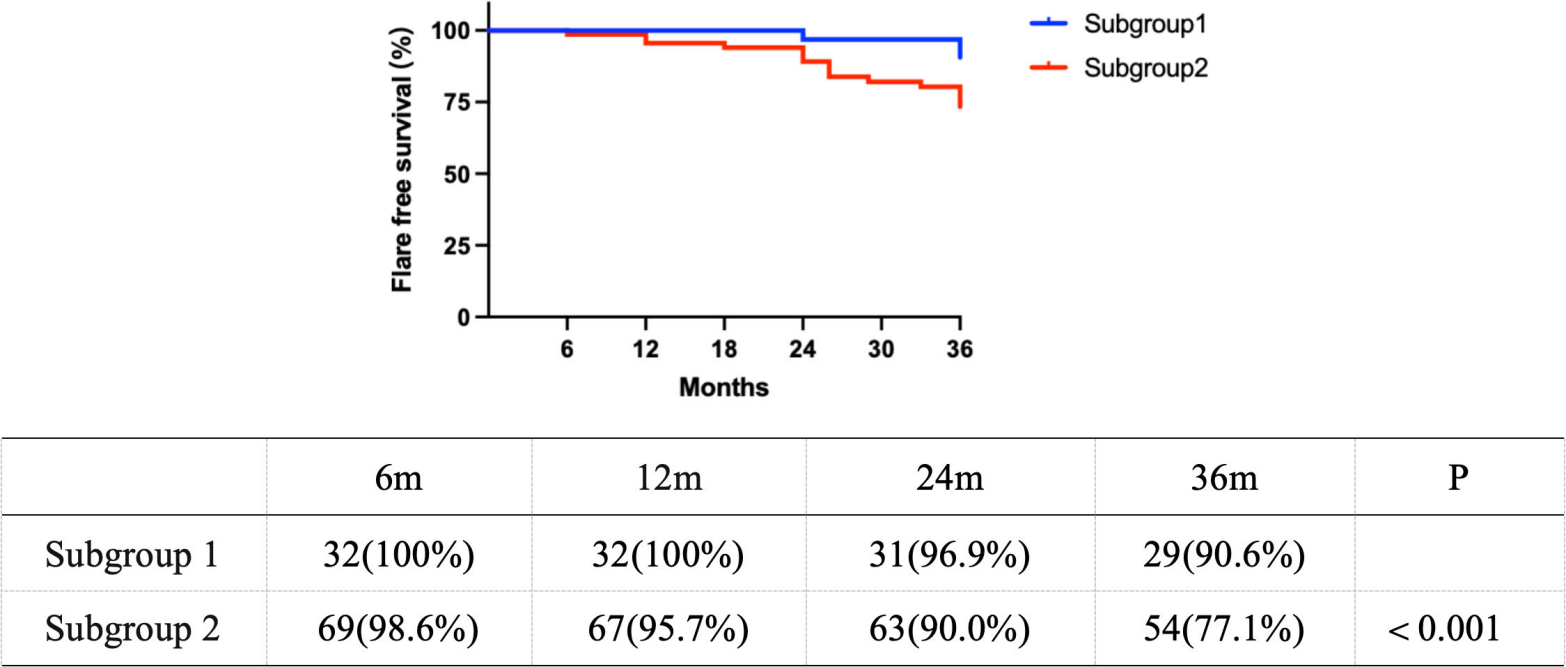
Kaplan–Meier curves comparing flare-free survival between subgroup 1 and subgroup 2.

After excluding events with infection-precipitated flares or treatment non-adherence, Kaplan–Meier analysis revealed a significantly lower flare-free survival in subgroup 2 compared to subgroup 1 (*p* < 0.01) (**Figure 2**). At 24 and 36 months, the flare risk was 2.5-fold higher in subgroup 2.

## Discussion

Accurate assessment of disease status and prognosis is crucial for managing cSLE. This study aimed to stratify cSLE patients into serologically distinct subsets using routinely measured autoantibodies. We found that patients positive for anti-Sm/RNP antibodies exhibited significantly higher disease activity and greater organ damage than their negative counterparts. The key new findings are the concurrent associations of this high-risk subgroup (the anti-Sm/RNP-positive subgroup) with specific *HLA-DRB1* risk alleles (DRB1*15:01, *15:02, *09:01) and a heightened interferon-alpha (IFN-α) signature.

The two-step cluster analysis provides hypothesis-defining subgroups rather than definitive disease classifications. Nevertheless, this exploratory approach offers meaningful clinical utility. In the study, two subgroups were identified by two-step cluster analysis, which were dominated by positive anti- Sm/RNP(subgroup 2) and by negative anti-Sm/RNP(subgroup 1), respectively, in line with our prior research (3). Two important organ impairments (LN and NPSLE) and higher SLEDAI scores were found at diagnosis in the positive anti-Sm/RNP group. This finding may be related to the impact of higher positive ratio of anti-dsDNA, anti-histone, anti-nucleosome, and anti-ribosomal P protein in the positive anti-Sm/RNP group. Herein, we further found that belimumab was required more frequently in the positive anti-Sm/RNP group. This was consistent with the high disease activity and the involvement of important organs in this group. From a practical point of view, our autoantibody-based subgroup leverages routinely available autoantibody data, offering a practical and efficient method for subgrouping cSLE patients with SLE.

The susceptibility loci for SLE detected by genome-wide association studies were mostly located in the major histocompatibility complex region, and *HLA-DRB1* polymorphisms, such as HLA-DRB1*15, were found associated with risk of SLE worldwide (9). However, analysis of *HLA-DRB1* in cSLE subgroups was rare. In our study, the HLA-DRB1*15 allele (DRB1*15:01, DRB1*15:02) and the HLA-DRB1*9 allele (DRB1*09:01) were more frequent in the positive anti-Sm/RNP group with higher disease activity and higher rates of important organ damage and flare. Our results were correspondent with the previous study that HLA-DRB1*15:01 and DRB1*09:01 were the prominent alleles associated with SLE in east Asia population, and DRB1*09:01 was significantly associated with the appearance of the anti-Sm antibody (10, 11). Meanwhile, HLA-DRB1*15 was also dominant in the SLE subgroup with a higher prevalence of nephritis in the Swedish and USA populations (2). The presence of anti-U1RNP antibodies was also found to be associated with *HLA-DRB1∗15* allele in a Polish mixed-connective tissue disease cohort (12). On the other hand, we found that the HLA-DRB1*8 allele(DRB1*8:03), the HLA-DRB1*11 allele (DRB1*11:01) and the HLA-DRB1*12 allele (DRB1*12:02) were more frequent in the negative anti-Sm/RNP group compared with the positive anti-Sm/RNP group. This was consistent with the previous result that DRB1*12:02 is negatively associated with LN in SLE patients (13). The pathological mechanism of intergroup differences of *HLA-DRB1* may be related to the peptide binding motif and T-cell receptor repertoire selection (10). Moreover, *HLA-DRB1* was involved in epigenetics, which may affect autoantibodies and disease spectrum. Specifically, HLA-DRB1*15:01 was found to be associated with hypomethylation, and the subsequent increased expression of *HLA-DRB1* in monocytes may contribute to the risk of autoimmune diseases like multiple sclerosis (14). Therefore, the subgroups of cSLE in this study had a basis in the *HLA-DRB1* genetic background.

To further address SLE heterogeneity, we compared the cytokine levels between two subgroups of patients with cSLE. Apart from HLA, the most strongly SLE-associated gene, type I IFN-responsive genes convey a contribution to SLE risk by enhancing signaling and activation of auto-reactive B cells. We found that IFN-α levels were significantly higher in subgroup 2 than in subgroup 1. This was consistent with the previous study that SLE patients possessing high levels of IFN-α were inclined to manifest more severe disease syndromes (15). Type I IFNs are produced in response to foreign material invasion for promoted maturation of dendritic cells and production of proinflammatory cytokines, leading to stimulated Th1 polarization and B-cell activation (16). It should also be noted that although the differences did not reach statistical significance, the observed elevations in IL-6, IL-10, and IFN-γ in subgroup 2 were consistent with the findings of higher SLEDAI scores and more organ damage (including LN and NPSLE) in this subgroup. This was supported by recent studies that high levels of IL-6, IFN-γ, and IL-10 were detected in the blood of patients with LN (2). Furthermore, IL-6 could disrupt the blood–brain barrier and initiate neuroinflammatory processes (17).

Additionally, IL-4 levels were higher in subgroup 1 than subgroup 2. Existing research indicates that IL-4 contributes to lupus pathogenesis by reversing B-cell anergy through STAT6 signaling, which promotes B-cell receptor recycling to the cell surface (18). However, IL-4 also exhibits protective effects by counteracting Toll-like receptor 7–driven aberrant B-cell differentiation (19). This dual role may explain why the clinical phenotype in subgroup 1 was less severe than that in subgroup 2. Moreover, the IL-17A level was lower in subgroup 2 than subgroup 1 in our study. A previous study reported the circulating IL-17A level of lupus patients was higher than healthy controls (20). However, the role of IL-17A in lupus pathogenesis was not consistently reported (21). Our result corroborated most of the prior research that IL-17A levels were not correlated favorably with disease activity as defined by SLEDAI (22), and IL-17A inhibitors and knockdown of the IL-17 gene did not improve survival, glomerulonephritis, or autoantibody levels in mice (23). These previous failures of IL-17 blockade in both clinical and animal studies raise the question of whether it might only be effective in a specific subset of patients, such as subgroup 1, while showing limited efficacy in subgroup 2. Thus, our findings of differences in cytokine levels between the two subgroups further bridge the immunogenetic architecture and clinical heterogeneity in cSLE.

The higher rate of belimumab use in subgroup 2 failed to confer a better flare-free survival compared to subgroup 1. This seemingly paradoxical finding aligned with emerging reports of belimumab resistance (24) and may be explained by the following factors. First, subgroup 2 was enriched with anti-dsDNA and anti-ribosomal P antibodies, both of which were established predictors of increased renal flare risk in patients receiving belimumab (25). Second, as stated above, IFN-α levels were significantly higher in subgroup 2 than subgroup 1. A previous study reported that belimumab induced changes in the B-cell receptor repertoire, particularly in the unswitched memory B-cell subset, which may reduce its naïve-like characteristics, while the IFN signature remained unchanged (26). The persistence of symptoms and signs in some SLE patients despite belimumab administration may be mediated by an activated IFN signature (26). Notably, the efficacy of anifrolumab—a biologic agent targeting the type I interferon pathway—was recently demonstrated in a Spanish cSLE case after 6 months of belimumab therapy proved ineffective, although the renal involvement was mild in the case (27). While our study cannot establish causality, we hypothesize that the prominent IFN-α signature in the positive anti-Sm/RNP subgroup might represent a factor contributing to a suboptimal response to B-cell–directed therapy. This hypothesis merits further studies to assess treatment response. It also should be acknowledged that subgroup 2 had higher SLEDAI scores and more severe organ damage at baseline, which represents a potential confounding factor when interpreting the lower flare-free survival despite more frequent belimumab use.

Furthermore, some clinical evidence indicated that the efficacy and safety of anifrolumab were comparable whether it was administered as a first-line biologic or after switching from belimumab (28). Therefore, for patients with high IFN-α levels who experience relapse or exhibit a suboptimal response to belimumab, switching to anifrolumab may be a potential therapeutic strategy (29, 30). To implement precision medicine for cSLE patients, further research is necessary to identify distinct subgroups of patients who could derive a greater benefit from specific biologic agents.

The limitations of our study were as follows. First, the size of our cohort was small. We will increase the sample size in future studies. Second, quantitative detection through ELISA will be applied, and autoantibody titers will be gathered and analyzed. Third, due to ethical and financial reasons, specimens of *HLA-DRB1* of normal controls were not collected. Instead, the *HLA-DRB1* frequency data for the general population were obtained from the public Allele Frequency Net Database (www.allelefrequencies.net), and the frequency of *HLA-DRB1* in SLE was higher than that in public databases. Furthermore, the findings of this study are supported and validated by existing *HLA-DRB1* data from lupus patients and healthy controls (2). Fourth, subgroup 2 had greater baseline disease severity, which represents a key indication for biologic therapy. This introduces potential confounding by indication when interpreting the lower flare-free survival despite more frequent belimumab use. Although we excluded infection-precipitated flares and non-adherence cases from the survival analysis, residual confounding by indication cannot be entirely ruled out. Additionally, the mechanisms driving the differences between the identified subgroups require further investigation. Nevertheless, our study shed light on better understanding of cSLE subtypes.

## Conclusion

In conclusion, our study demonstrates that stratification of cSLE patients based on routinely available autoantibody data is not only feasible but also uncovers two patient subgroups with profound differences. The anti-Sm/RNP positive subgroup (subgroup 2) encapsulates a more severe disease phenotype, marked by greater organ damage at onset, a distinct genetic predisposition conferred by HLA-DRB1*15 and *09 alleles, and high IFN-α signature. Our study bridges the gap between serologically defined subgroups and underlying immunopathogenesis, offering a rationale for treatment strategies.

## Data Availability

The raw data supporting the conclusions of this article will be made available by the authors, without undue reservation.

## Supplementary material

Supplementary Figure 1 **(A)** Summary and quality assessment of the two-step cluster analysis model. The model was constructed using the two-step algorithm with the nine autoantibodies as input variables, which yielded two clusters. The validity was evaluated using the silhouette coefficient (value >0.50 was interpreted as good fitting, between 0.30 and 0.50 as fair, and <0.30 as poor). The silhouette coefficient of 0.4 indicated a fair quality model. **(B)** Autoantibody profile comparison between the two cSLE subgroups identified by cluster analysis. The dot plot visualized the positive (pos) or negative (neg) state of the nine autoantibodies within subgroup 1 and subgroup 2. **(C)** Predictor importance ranking of the nine autoantibodies in the cluster model. Anti-U1RNP and anti-Sm antibodies were the most important predictors for distinguishing the subgroups.
