# Supplementary figures and images for: Subgroups based on autoantibody status associated with clinical manifestations, HLA-DRB1 variants, cytokines, and flare of vasculitis in childhood-onset systemic lupus erythematosus
Source: Front Immunol. 2026 Mar 16;17:1766478. doi: 10.3389/fimmu.2026.1766478 (PMC13033651; doi:10.3389/fimmu.2026.1766478)

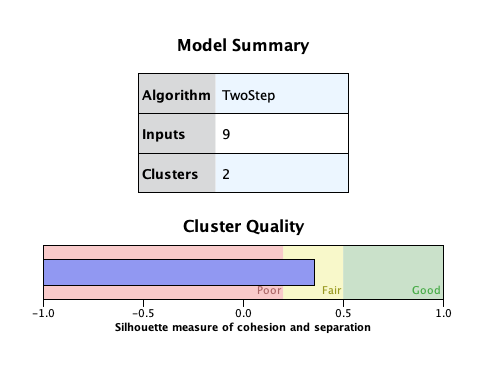

Supplement: Supplementary Figure 1 — (A) Summary and quality assessment of the two-step cluster analysis model. The model was constructed using the two-step algorithm with the nine autoantibodies as input variables, which yielded two clusters. The validity was evaluated using the silhouette coefficient (value >0.50 was interpreted as good fitting, between 0.30 and 0.50 as fair, and <0.30 as poor). The silhouette coefficient of 0.4 indicated a fair quality model. (B) Autoantibody profile comparison between the two cSLE subgroups identified by cluster analysis. The dot plot visualized the positive (pos) or negative (neg) state of the nine autoantibodies within subgroup 1 and subgroup 2. (C) Predictor importance ranking of the nine autoantibodies in the cluster model. Anti-U1RNP and anti-Sm antibodies were the most important predictors for distinguishing the subgroups. [file Image1.jpeg]

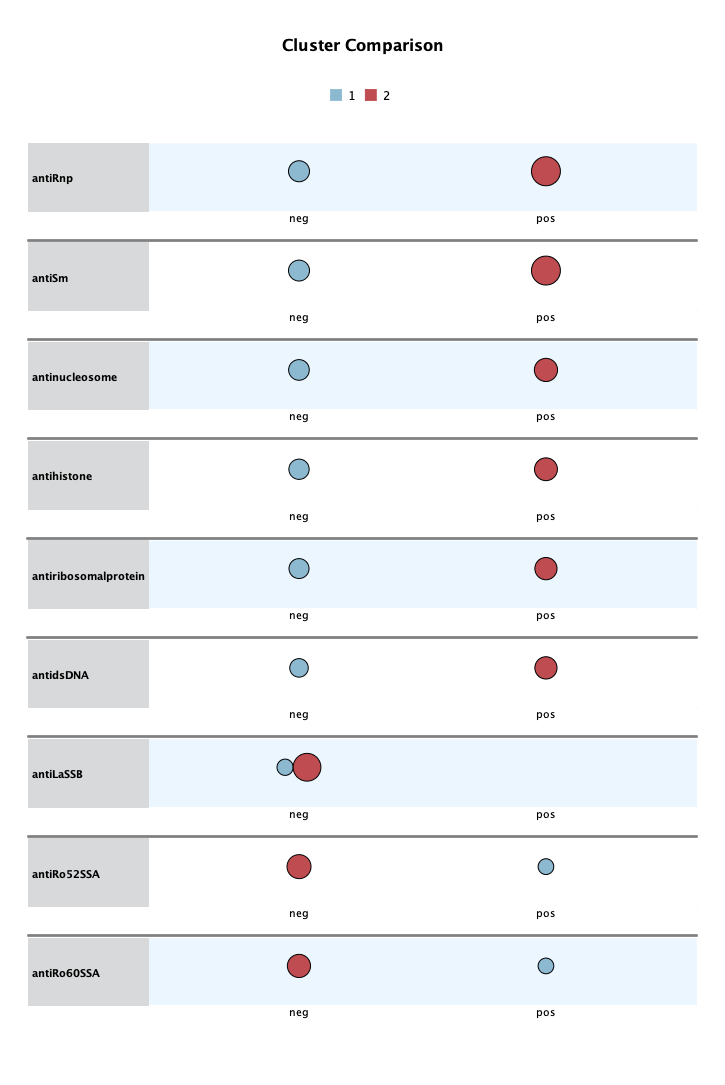

Supplement: Supplementary file 2 [file Image2.jpeg]

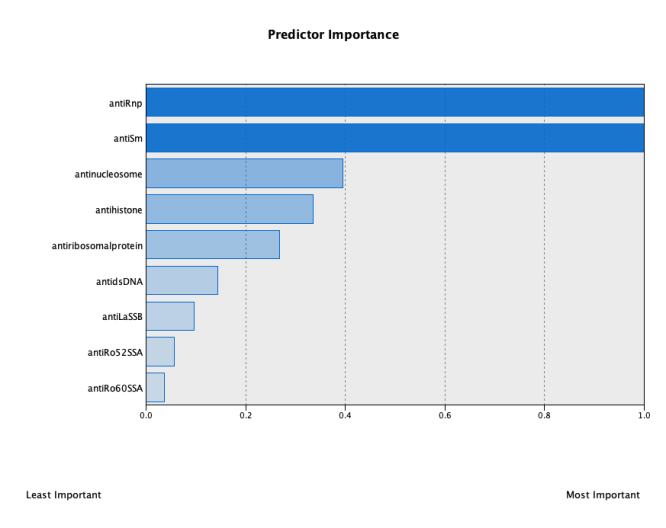

Supplement: Supplementary file 3 [file Image3.jpeg]
